# Identification of an autophagy-related gene signature for survival prediction in patients with cervical cancer

**DOI:** 10.1186/s13048-020-00730-8

**Published:** 2020-11-07

**Authors:** Hengyu Chen, Qingchun Deng, Wenwen Wang, Huishan Tao, Ying Gao

**Affiliations:** 1 Department of Pancreatic Surgery, Union Hospital, Tongji Medical College, Huazhong University of Science and Technology, Wuhan, 430022 China; 2 NHC Key Laboratory of Hormones and Development, Tianjin Institute of Endocrinology, Tianjin Medical University Chu Hsien-I Memorial Hospital, Tianjin, 300070 China; 3 Department of Gynecology, The Second Affiliated Hospital of Hainan Medical University, Haikou, 570102 China; 4 Department of Gynecology and Obstetrics, Union Hospital, Tongji Medical College, Huazhong University of Science and Technology, Wuhan, 430022 China

## Abstract

Cervical cancer is one of the most common female malignancy that occurs worldwide and is reported to cause over 300,000 deaths in 2018. Autophagy controls the survival and death of cancerous cells by regulating the degradation process of cytoplasm and cellular organelle. In the present study, the differentially expressed autophagy-related genes (ARGs) between healthy and cancerous cervical tissues (squamous cell neoplasms) were obtained using data from GTEx and The Cancer Genome Atlas (TCGA) database. The functionalities of the differentially expressed ARGs were analyzed using Gene Ontology (GO) as well as the Kyoto Encyclopedia of Genes and Genomes (KEGG) database. Next, we conducted univariate Cox regression assay and obtained 12 ARGs that were associated with the prognosis of cervical cancer patients. We carried out a multivariate Cox regression analysis and developed six ARG-related prognostic signature for the survival prediction of patients with squamous cell cervical cancer (Risk score = − 0.63*ATG3–0.42*BCL2 + 0.85*CD46–0.38*IFNG+ 0.23*NAMPT+ 0.82*TM9SF1). Following the calculation of risk score using the signature, the patients were divided into high and low-risk groups according to the median value. Kaplan-Meier curve demonstrated that patients with a high-risk score tend to have a poor prognosis (*P* < 0.001). The value for area under the curves corresponding to the receiver operating characteristic (ROC) was 0.740. As observed, the expression of IFNG was negatively associated with lymph node metastasis (*P* = 0.026), while a high-risk score was significantly associated with increased age (*P* = 0.008). To further validate the prognostic signature, we carried out a permutation test and confirmed the performance of the risk score. In conclusion, our study developed six ARG-related prognostic signature for patients with squamous cell cervical cancer, which might help in improving the prognostic predictions of such patients.

## Introduction

Cervical cancer is one of the challenging malignancies observed among females worldwide. Over 300,000 women die of cervical cancer each year, and approximately 90% of them are from low- to middle-income countries [1]. Infection of high-risk human papillomavirus (HPV) is one of the main reasons for cervical cancer, although HPV infection cannot fully elucidate the occurrence of cervical cancer [2]. Five-year overall survival (OS) of locally advanced cervical cancer is around 70% following chemotherapy [3]. Survival prediction of cervical cancer patients mainly depends on FIGO stages. However, a simple and sensitive detection method for predicting prognosis still needs to be developed. Autophagy is defined as the process by which cells self-degrade to maintain homeostasis under stress. The dysregulation of autophagy is involved in various kinds of cancers [4]. Recent studies showed that autophagy has emerged as an essential system in tumorigenesis. It can suppress or promote tumor progression, i.e., its role can also be neutral. In gynecological cancers, decreased levels of autophagy could promoted the initiation of early-stage cancer [5], while an increase of autophagy levels could promote tumor cell survival under nutrient-deficient microenvironment [6]. Previous studies investigated the role of autophagy in cervical cancer. Zhu et al. reported that autophagy-related genes (ARGs), Beclin-1, and LC3 were downregulated in the early stages of cervical cancer [7]. High expressions of Beclin-1 and LC3 were associated with poor prognosis in early-stage cervical squamous cell carcinoma. Xu et al. investigated that the inhibition of autophagy could improve cisplatin chemotherapy sensitivity in HeLa cervical cancer cells [8]. These findings indicated that autophagy was tightly associated with the progression of cervical cancer, and ARGs could serve as promising therapeutic targets for cervical cancer patients.

The most common approach to predict the prognosis of cervical cancer patients is based on the FIGO system. However, FIGO staging is not accurate enough for assessing prognosis. Typically, the prognosis of patients with the same FIGO stage shows considerable heterogeneity in cervical cancer. Thus, an advanced system for individualized prognosis prediction is urgently needed. Recently, gene expression signature-based on ARGs has been constructed to predict the prognosis of various kinds of cancers, including colon cancer [9], ovarian cancer [10], breast cancer [5], and glioblastoma [11]. ARG-related signature that could predict the survival of cervical cancer has not been investigated yet. In the present study, we constructed an ARG-related prognosis signature for squamous cell cervical cancer patients using gene expression profile data of TCGA and GTEx database. The performance of the signatures was validated by the Kaplan-Meier curve, the area under the curve (AUC), and the permutation test. Functional enrichment analysis was also carried out to investigate the function of ARGs associated with cervical cancer prognosis. Our study established a novel prognostic model for cervical cancer, which accurately predicted the prognosis of squamous cell cervical cancer.

## Materials and methods

### TCGA, GTEx and GEO dataset

Gene expression quantification data and clinical information of cervical cancer patients were downloaded from TCGA. Clinical data and expression data of genes in healthy cervix tissue were obtained from the GTEx database. Autophagy genes were extracted from the Human Autophagy Database (HADb). Samples with more than 25% of ARG expressions with or without clinical data were excluded from the study. Gene array data were combined and normalized using the R Bioconductor limma package. Overall, a total of 255 cervical cancer samples and 12 healthy cervix controls were enrolled in the present study. GSE44001 was used as a validation dataset. Gene expression and clinical data of patients in GSE44001 were downloaded from the GEO database. Ethics committee approval was not necessary as the data were downloaded from public databases.

### Data processing

R Programming language was the principal tool for analyzing data throughout the study. The differentially expressed ARGs between cervical cancer samples and healthy controls were obtained using the EdgeR package, whereas |log FoldChange| (|logFC|) > 2 was selected as differentially expressed ARGs. The bar plot was constructed by ggpubr package. GO and KEGG analyses were conducted by the enrichplot package. Survival analysis and survival curves were done by the survival and survminer packages. SurvivalROC package was used to create a ROC curve, which was used to evaluate the sensitivity and specificity of the risk score. Nomogram was generated according to the ARG related signature and traditional clincal prognostic factors using rms package. The version of R Bioconductor used in the present study was R 3.6.2.

### Statistical analysis

The differential expressed ARGs were selected based on an adjusted *P* value< 0.05 (Benjamini & Hochberg correction). Univariate Cox regression analysis was performed to select ARGs associated with OS, and multivariate Cox regression was carried out to select candidate prognostic genes to construct risk score signature. Kaplan-Meier curve was used to assess differences in OS between high-risk score group and low-risk score group. Hazard ratios (HR) and 95% confidence intervals (CI) were calculated to evaluate variables associated with OS. X^2^ test was used to detect differences in parameters associated with OS between high-risk groups and low-risk groups. *P* < 0.05 was used as a cut off value for significance.

## Results

### Differentially expressed ARGs between squamous cell cervical cancer samples and healthy controls

**Fig. 1:**
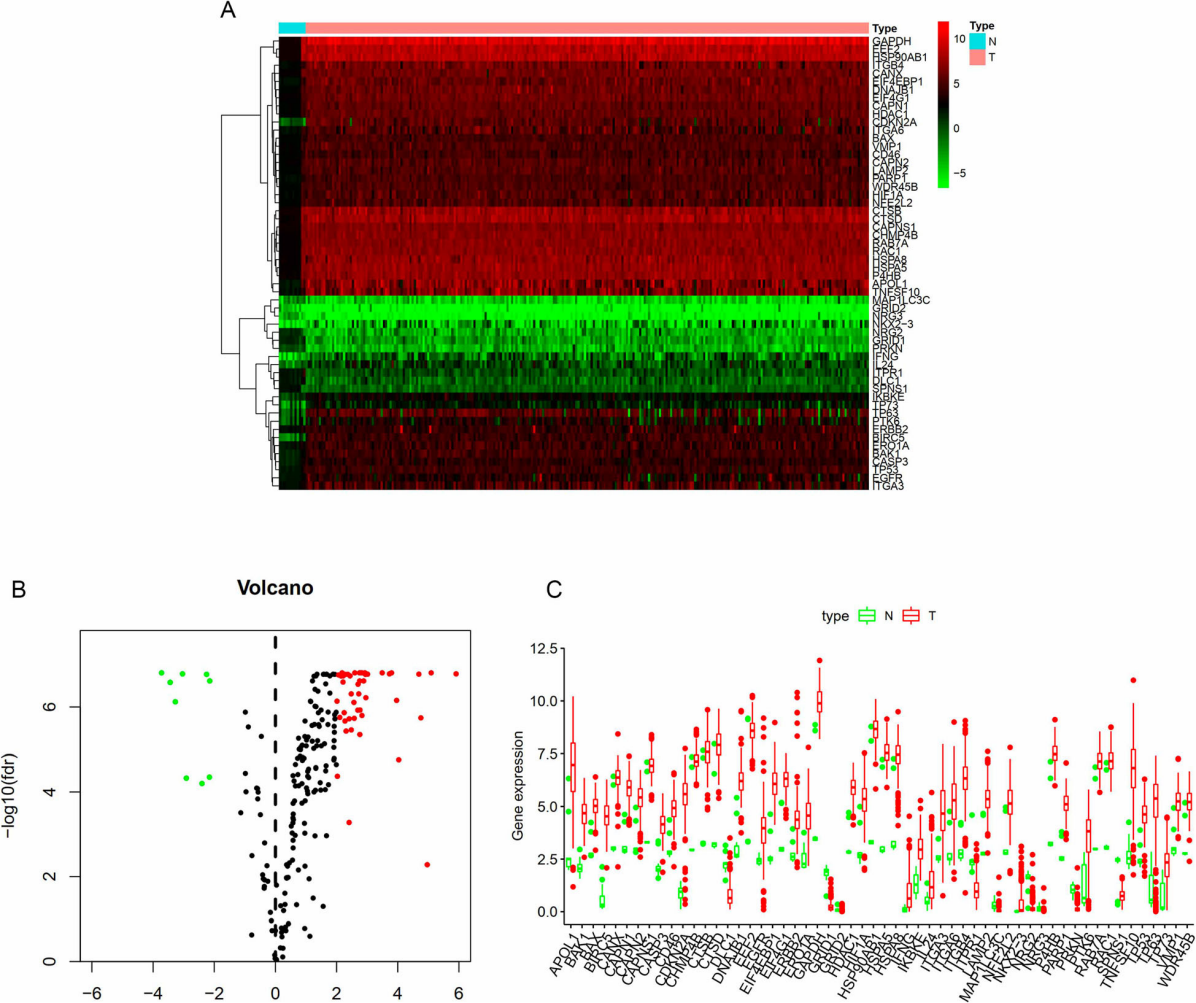
Differentially expressed ARGs between squamous cell cervical cancer patients and normal controls. **a** Heatmap showed gene expression of 56 differentially expressed ARGs between normal cervical tissues (N) and cervical cancer tissues (T). Red color represents for high expression while green color indicates low expression. **b** Volcano plot of differentially expressed ARGs. Red plots indicate genes are upregulated in cervical cancer samples with logFC> 2. Green plots indicate genes are downregulated in cervical cancer samples with logFC<− 2. **c** The boxplot of differentially expressed ARGs. N: Normal cervical tissue. T: Cervical cancer tissue

Two hundred thirty-four genes directly or indirectly involved in autophagy were obtained from the HADb database. The expression of 234 ARGs was extracted from 255 cervical cancer patients and 12 standard cervix control samples. A total of 56 differentially expressed genes were identified, as shown in Fig. 1a-b. Among them, 9 genes were significantly downregulated in cervical cancer samples, while 47 genes were upregulated. Boxplot in Fig. 1c was used to visualize the expression differences of 56 genes in tumor and standard samples.

### Bio-functional analysis of identified differentially expressed ARGs in squamous cell cervical cancer

**Fig. 2:**
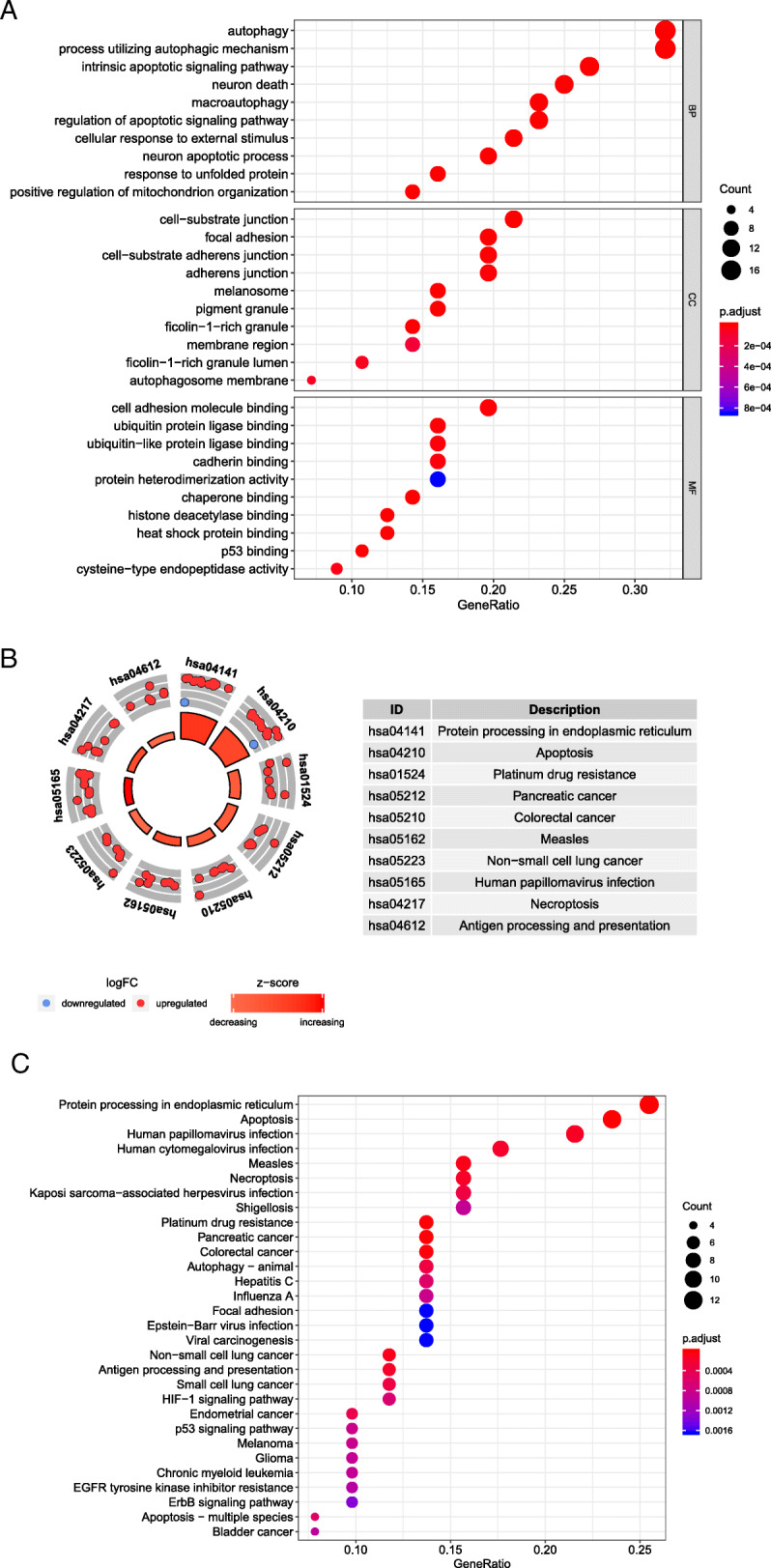
GO and KEGG analysis of differentially expressed ARGs. **a** The bubble plot of GO functional enrichment analysis. BP: Biological Process. CC: Cellular Components. MF: Molecular Function. **b** The circle plot of KEGG pathway enrichment analysis. Red plots indicate upregulated genes in the pathway while blue plots indicate downregulated genes. Higher Z-value means higher expression of enriched pathways. **c** The bubble plot of KEGG analysis

To further investigate the biological function of differentially expressed ARGs, we conducted GO and KEGG analysis. The results were shown in Fig. 2a and b. For GO analysis, in the term of biological process (BP), the targeted genes were highly enriched in autophagy, intrinsic apoptotic signaling pathway, and cellular response to external stimulus. This process was highly related to cancer development. For the cellular component (CC) part, the process associated with cervical cancer development, including cell-substrate junction and focal adhesion were significantly enriched by these genes. In the term of molecular function (MF), genes were highly related to p53, cell adhesion molecule, and cadherin bindings, respectively. For KEGG analysis, the differentially expressed ARGs were enriched in essential pathways associated with cancer progressions, such as apoptosis pathway, platinum drug resistance pathway, pathways related to pancreatic cancer, colorectal cancer, and non-small cell lung cancer.

### Construction of autophagy-related gene signature in squamous cell cervical cancer

**Fig. 3:**
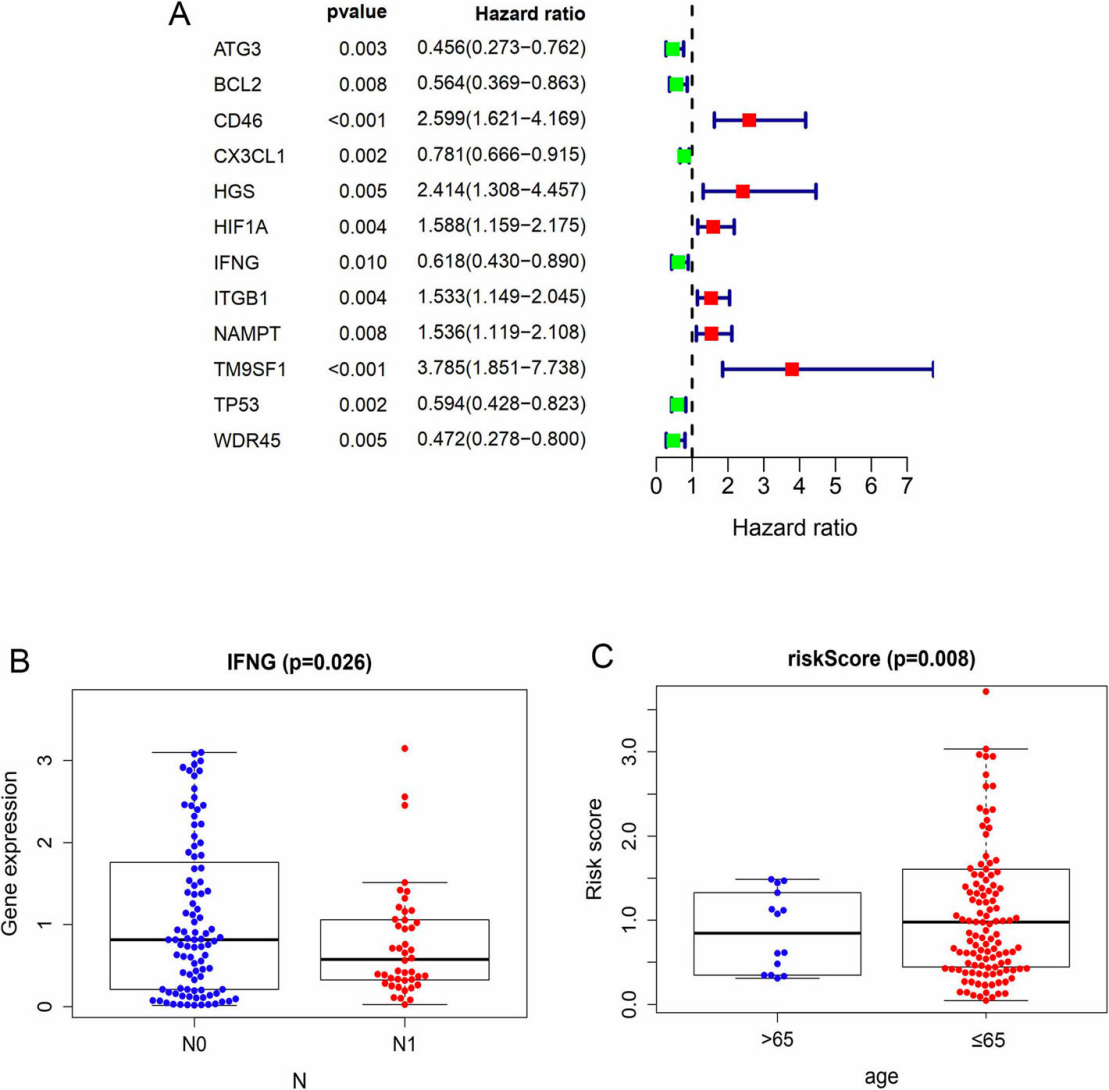
ARGs related to overall survival (OS) in squamous cervical cancer. **a** ARGs related to OS were identified using Univariate Cox regression analysis. **b** IFNG expression was reversely related to N stage in cervical cancer. **c** Risk score signature is significantly associated with age in cervical cancer patients

**Fig. 4:**
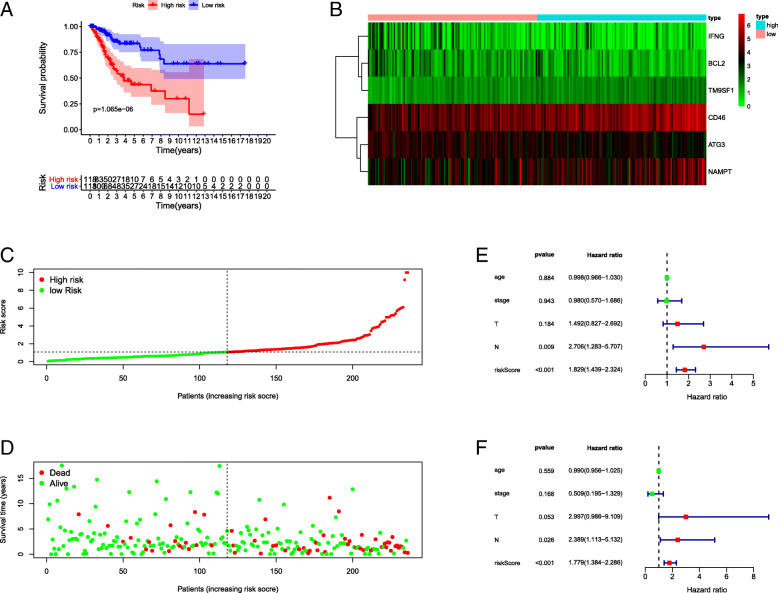
Performance of the ARGs signature in TCGA-CESC dataset. **a** Kaplan-Meier curve showed that high risk score group had shorter OS than low risk score group. **b** The heatmap of the expression of 6 ARGs in the signature in low risk score group and high risk score group. **c** The number of patients in different risk score groups. **d** Survival time and survival status in patients with different risk scores. Increasing risk score was associated with shorter survival time and more deaths. **e** Univariate Cox regression analysis identified clinical factors associated with OS in TCGA dataset. Higher risk score and higher N stage were significantly associated with poor prognosis. **f** Multivariate Cox regression analysis identified clinical factors independently associated with OS. Risk score and N stage were independent prognostic factors for cervical cancer

To construct the risk score signature using differentially expressed ARGs, we first conducted univariate Cox regression analysis to select candidate ARGs associated with OS in cervical cancer patients. As shown in forest plots of Fig. 3a, 12 survival related ARGs included ATG3, BCL2, CD46, CX3CL1, HGS, HGS, HIF1A, IFNG, ITGB1, NAMPT, TM9SF1, TP53, and WDR45. Next, we carried out multivariate Cox regression analysis and identified 6 genes (ATG3, BCL2, CD46, IFNG, NAMPT, TM9SF1) that were independently associated with OS in squamous cell cervical cancer patients (Fig. 4). Among them, high expressions of CD46, NAMPT, and TM9SF1 were associated with unfavorable prognosis, while high expression of ATG3, BCL2, and IFNG were associated with favorable prognosis. Our prognostic model constructed using 6 ARGs is as follows: Risk Score = (− 0.634 * expression of ATG3) + (− 0.425 * expression of BCL2) + (0.849 * expression of CD46) + (− 0.379 * expression of IFNG) + (0.233 * expression of NAMPT) + (0.826 * expression of TM9SF1). We further analyzed the relationship between the signatures and clinicopathologic variables. In this respect, we identified that the signature was significantly associated with age (*P* = 0.008), and the expression of IFNG was different among N stages (*P* = 0.026) (Fig. 3b-c) for most signature related genes.

### Performance of autophagy-related gene signature in TCGA-CESC dataset

**Fig. 5:**
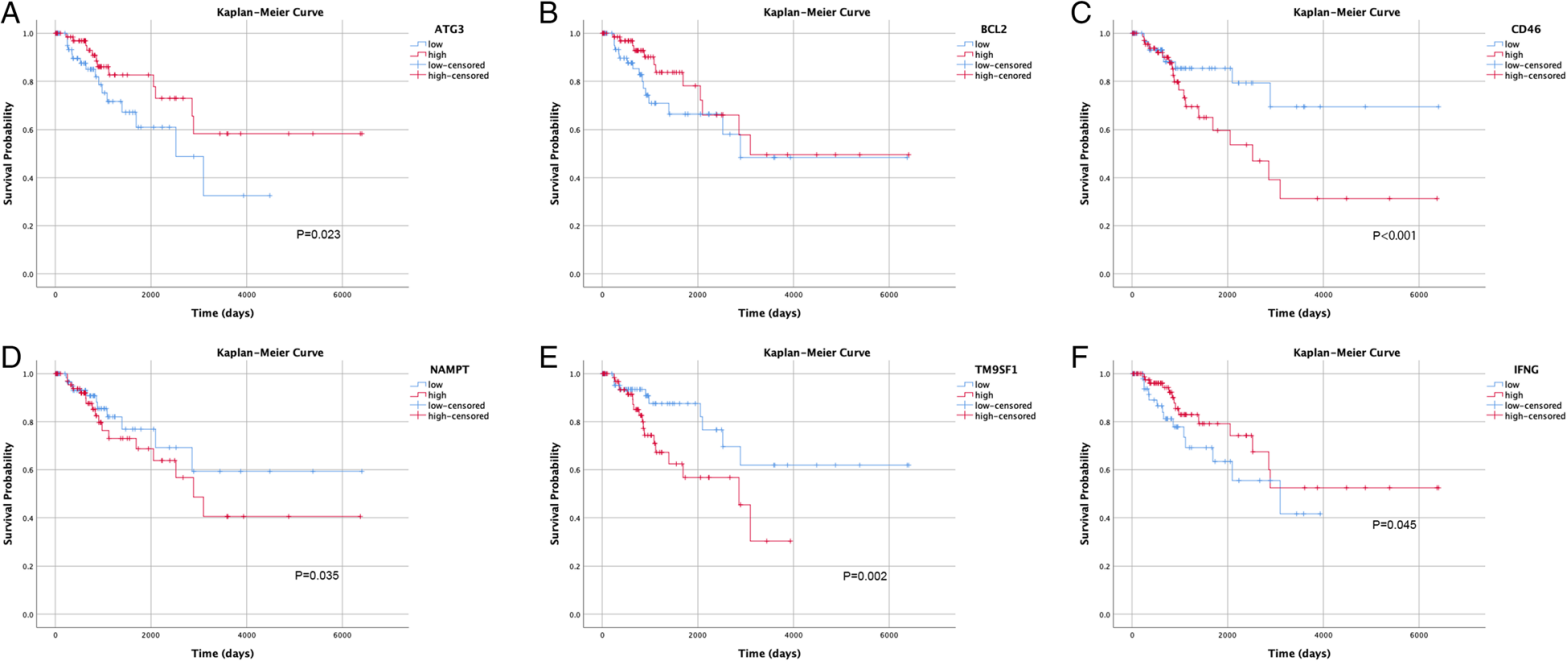
Kaplan-Meier Curves for genes in the signature. The correlation between the expression of **a** ATG, **b** BCL2, **c** CD46, **d** NAMPT, **e** TM9SF1 and **f** IFNG and OS was observed in TCGA dataset

**Fig. 6:**
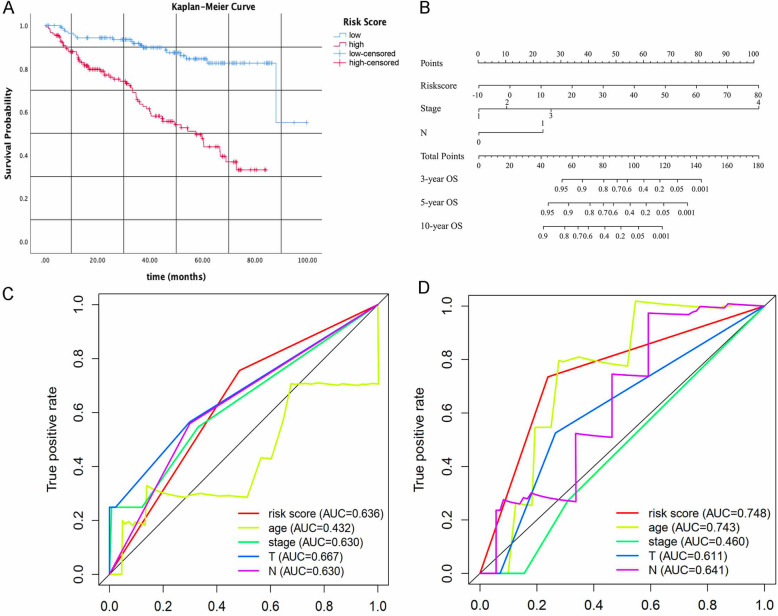
Validation of the 6 ARGs signature in GSE44001. **a** Kaplan-Meier curve indicated that higher risk score was associated with unfavorable prognosis in GSE44001 dataset. **b** Prognostic nomogram for squamous cell cervical cancer patients. **b-c** The ROC curve of OS for 6 ARGs signature and clinical factors in GSE44001 and TCGA datasets

We calculated the risk score for each patient in the TCGA-CESC dataset based on which, the patients were divided into high-risk and low-risk score groups according to the median. As shown in Fig. 5a, the OS of patients with higher risk scores was significantly shorter than patients with lower risk scores (*P* = 1.065e-06). The relationship between signature-related genes and different risk score groups is shown in the heatmap (Fig. 5b). Also, the survival time and status of such patients were displayed depending on their risk score (Fig. 5c-d). In order to confirm whether the risk score signature was independently associated with OS, we carried out univariate and multivariate Cox regression analysis using clinical factors such as age, tumor stage, T stage, and N stage. As shown in Fig. 5e, the N stage and risk score signature were significantly associated with OS, as observed in a univariate Cox regression assay. These two factors were also included in the multivariate Cox regression assay. As shown in Fig. 5f, the risk score was identified as an independent risk factor for OS with HR = 1.779 (1.384–2.286), *P* < 0.001. Furthermore, a nomogram was generated based on the ARGs risk score signature and traditional clinical prognostic factors (Fig. 6b).

### Validation of autophagy-related gene signature in an independent cohort GSE44001

To further confirm the accuracy of the 6 ARGs signature in predicting prognosis, we utilized gene expression and clinical data in the GSE44001 dataset from the GEO database. A total of 300 patients were divided into high-risk groups (*n* = 153) and low-risk groups (*n* = 147) according to the risk score median used in the TCGA database. Again, the signature could successfully predict OS in this cohort (*P* < 0.001) (Fig. 6a). ROC curve was also used to evaluate the accuracy and specificity of the risk score signature in both the TCGA GSE44001 dataset. As shown in Fig. 6c-d, the AUC of the signature in GSE44001 and TCGA dataset was 0.636 and 0.748, respectively, which indicated that the signature could be used as a valuable tool in predicting prognosis of squamous cell cervical cancer.

## Discussion

The incidence of cervical cancer in middle and low-income countries is still high. Despite advances in treatment during the past decades, cervical cancer is still a heavy burden for medical resources and patients in these countries. The most common prognosis prediction system for cervical cancer is the FIGO (International Federation of Gynecology and Obstetrics) staging system [12]. Although FIGO staging is considered to be critical for the determination of prognosis in cervical cancer patients, the clinical outcomes of squamous cell cervical cancer can vary among patients with the same FIGO stage. Recently, many computational models were constructed utilizing mRNA [13], miRNA [14], or lncRNA [15] expression data from the public database to predict the prognosis of cervical cancer patients. The idea that autophagy could serve as a potential therapeutic target in cancer has been studied for several years [16]. Besides, numerous autophagy-related signatures were developed to predict prognosis in various types of cancer [17–19]. In the present study, we utilized a high-throughput gene expression data in the TCGA-CESC dataset to construct an ARG signature; the signature showed great ability in predicting the prognosis of squamous cervical cancer patients. Furthermore, the prognostic value was confirmed by an independent GEO dataset (GEO44001). In our study, the differentially expressed ARGs between cervical cancer patients, and healthy controls were subjected to GO and KEGG analysis. The GO results showed that differentially expressed ARGs were significantly enriched in autophagy, and intrinsic apoptosis signaling pathway in terms of biological processes, such as focal adhesion, the cell-substrate junction, cellular adhesion molecule binding, cadherin binding, and P53 binding (in terms of molecular function). These results suggested that differentially expressed ARGs were highly correlated with the cancer progression process. Also, the KEGG analysis indicated that the differentially expressed ARGs were involved in cancer development, which revealed that ARGs could serve as potential biomarkers or therapeutic targets for cervical cancer. After univariate and multivariate Cox regression assay of differentially expressed ARGs, 6 ARG (ATG3, BCL2, CD46, IFNG, NAMPT, TM9SF1) related prognostic signatures for squamous cell cervical cancer were constructed. ATG3 was observed to be upregulated in various kinds of tumor tissues, which played an oncogenic role in colon cancer [20] and breast cancer [21]. ATG3 is reported to serve as a tumor protective factor in general, and the mechanisms of its oncogenic function are tissue-specific. Moreover, miR-431-5p is reported to decrease the expression of ATG3 in colon cancer. Therefore, the dysregulation of miR-431-5p expressions can trigger colon cancer progression [20]. LncRNAs with an oncogenic function such as lncRNA-HOTAIR, lncRNA-PVT1, and lncRNA-GAS5 can stabilize ATG3 mRNA and increase its expression in cancer cells [21–23]. In the present study, we identified ATG3 as a favorable prognostic factor in cervical cancer. BCL2 is an antiapoptotic factor, and overexpression of BCL2 is reported to be associated with poor prognosis in diffuse large B cell lymphoma. Such an observation indicated the occurrence of acute myeloid leukemia with FLT3 mutations [24]. In breast cancer, the prognostic role of BCL2 is still controversial. Bouchalova et al. reported that overexpression of BCL2 is a favorable prognostic marker in triple-negative breast cancer (TNBC) [25] while Ozretic et al. argued that elevated expression of BCL2 is an independent prognostic factor for an unfavorable prognosis in TNBC [26]. Also, Eom et al. reported that the prognostic role of BCL2 is subtype-specific and only considered as an excellent prognostic marker in luminal breast cancer [27]. In the present study, we found out that BCL2 favored prognostic factor in squamous cervical cancer, which meant that a higher expression of BCL2 was associated with a lower risk score. CD46 is reported to predict unfavorable prognosis in ovarian cancer [28] and breast cancer [29] as it belongs to type I membrane protein, and therefore, it can provide cell protection against autologous complement. In cervical cancer, the inhibition of CD46 is reported to enhance complement-dependent cytolysis in cervical cancer cell line ME180 [30]. In consistent with the previous finding, our results suggested that CD46 is an independent prognostic factor for poor prognosis in cervical cancer. IFNG is an immune response gene, wherein some single nucleotide polymorphisms (SNPs) are involved in HPV-initiated cervical carcinogenesis [31]. Also, gene-gene interaction between CD28 and IFNG could increase females’ susceptibility to cervical cancer [32]. Our study revealed that the expression of IFNG is reversely correlated with the risk score, which suggested that IFNG could play a decisive prognostic role in squamous cervical cancer. Interestingly, high expression of IFNG is reversely correlated with N stage, indicating complicated roles of IFNG in cervical cancer progression. NAMPT is a family member of nicotinic acid phosphoribosyltransferase (NAPRTase). Although the functional and prognostic role of NAMPT has not been well studied in cervical cancer, it is reported to predict prognosis in breast invasive ductal carcinoma [33], glioblastoma [34] and colorectal cancer [35]. TM9SF1 plays an essential role in autophagy, and its expression is reported to have a significant role in AS associated prognosis prediction in cervical cancer patients [36]. In our study, high expression of TM9SF1 worsened prognosis in cervical cancer.

## Conclusion

In summary, this is the first study to investigate the prognostic role of autophagy genes in squamous cervical cancer. An autophagy-related gene signature was constructed to predict prognosis for cervical cancer patients. The signatures were validated in an independent cervical cancer dataset, which suggested that it has an excellent clinical value. The performance of the risk score signatures still needs to be tested in larger cohorts of squamous cervical cancer patients, and the investigation of the molecular mechanisms of genes in the signature is considered for future work.

## Data Availability

The datasets generated and/or analysed during the current study are available in the TCGA (https://portal.gdc.cancer.gov/), GTEx (https://www.gtexportal.org/home/index.html) and GEO repository (https://www.ncbi.nlm.nih.gov/geo/).
